# Characterization of DNA methylation clock algorithms applied to diverse tissue types

**DOI:** 10.18632/aging.206182

**Published:** 2025-01-03

**Authors:** Mark Richardson, Courtney Brandt, Niyati Jain, James L. Li, Kathryn Demanelis, Farzana Jasmine, Muhammad G. Kibriya, Lin Tong, Brandon L. Pierce

**Affiliations:** 1 Department of Public Health Sciences, University of Chicago, Chicago, IL 60615, USA; 2 Department of Medicine, University of Pittsburgh, Pittsburgh, PA 15261, USA; 3 Department of Human Genetics, University of Chicago, Chicago, IL 60615, USA; 4 Comprehensive Cancer Center, University of Chicago, Chicago, IL 60615, USA

**Keywords:** epigenetic aging, epigenetic clock, DNA methylation

## Abstract

Background: DNA methylation (DNAm) data from human samples has been leveraged to develop “epigenetic clock” algorithms that predict age and other aging-related phenotypes. Some DNAm clocks were trained using DNAm obtained from blood cells, while other clocks were trained using data from diverse tissue/cell types. To assess how DNAm clocks perform across non-blood tissue types, we applied DNAm algorithms to DNAm data generated from 9 different human tissue types.

Methods: We generated array-based DNAm measurements for 973 samples from deceased tissue donors from the GTEx (Genotype Tissue Expression) project representing nine distinct tissue types: lung, colon, prostate, ovary, breast, kidney, testis, skeletal muscle, and whole blood. For all samples, we generated DNAm clock estimates for 8 epigenetic clocks and characterized these tissue-specific clock estimates in terms of their distributions, correlations with chronological age, correlations of clock estimates between tissue types, and association with participant characteristics.

Results: For each clock, the mean DNAm age estimate varied substantially across tissue types, and the mean values for the different clocks varied substantially within tissue types. For most clocks, the correlation with chronological age varied across tissue types, with blood often showing the strongest correlation. Each clock showed strong correlation across tissues, with some evidence of some residual correlation after adjusting for chronological age. In lung tissue, smoking generally had a positive association with epigenetic age.

Conclusions: This work demonstrates how differences in epigenetic aging among tissue types leads to clear differences in DNAm clock characteristics across tissue types. Tissue or cell-type specific epigenetic clocks are needed to optimize predictive performance of DNAm clocks in non-blood tissues and cell types.

## INTRODUCTION

Changes in the human epigenome occur as humans age, and these epigenetic alternations are considered a hallmark (and a cause) of human aging [1]. One such epigenetic feature is DNA methylation (DNAm) at cytosine-guanine dinucleotides. DNAm plays a key role in gene regulation, particularly DNAm at CpG islands near gene promoters, which is indicative of gene silencing [2]. Early studies of aging and DNAm reported age-related changes in cancer cells [3, 4], human tissues [5], mouse tissues [6], as well as in blood samples from twin studies [7]. Subsequent studies identified regions enriched for aging-related DNAm changes, including promoters of polycomb group protein target genes [8] and bivalent chromatin domains [9]. Associations between age and DNAm at CpG sites across the human genome have been thoroughly characterized in many studies (most often based on DNAm data from blood cells) [2, 10, 11], and these age-related changes are likely accompanied by changes in other epigenetic features (e.g., histone modifications, nucleosome positioning, chromatin conformation) [8, 12]. While our understanding of these changes and their implications for cellular function is incomplete, the concepts of “loss of constitutive heterochromatin” and “epigenetic drift” have been used to describe how these changes relate to aging [12].

In human studies, DNAm data has often been generated using commercial arrays that measure DNAm at ~27,000 to ~850,000 CpG sites. These data have been used to develop DNA methylation (DNAm) clock algorithms that leverage data on many CpG sites to predict age and other aging-related phenotypes [2, 11, 13]. First generation DNAm clocks (i.e., Hannum clock and Horvath clock) were trained with the goal of accurately estimating chronological age of a biological sample. These clocks estimate acceleration of biological age (relative to chronological age), a measurement that has been shown to positively correlate with aging-related phenotypes [2]. Later generations of DNAm clocks (e.g., GrimAge [14], PhenoAge [15]) were trained on additional health- and aging-related variables (e.g., smoking, circulating biomarkers) in order to generate biological aging estimates that more strongly predict health and lifespan. Additional DNAm clocks of interest include “mitotic clocks”, such as EpiTOC (Epigenetic Timer of Cancer), which estimates the mitotic age of cells, how many stem cell divisions have occurred, and provides a subsequent estimate of cell age [16]. In recent years, clocks have been developed that use larger numbers of CpGs, and sophisticated variable selection methods, in order to improve prediction of chronological age, including Vijayakumar and Cho’s “EpiClock” (~7000 CpGs) [17], AltumAge (20,318 CpGs) [18], and the Zhang clock (514 CpGs) [19]. In addition, a clock that estimates “pace of aging” (DunedinPACE) was developed using two decades of longitudinal biomarker data (173 CpGs) [20].

The DNAm clocks developed to date were trained using either (1) DNAm obtained from blood cells only, or (2) DNAm obtained from diverse tissue types, but with the majority of training data coming from blood cells [11]. Thus, DNAm clocks may not perform equally well across tissue types, and few studies have compared the performance of DNAm aging clock algorithms across a variety of non-blood tissue types [21, 22]. It is important to understand clocks’ performance across different tissues, as clinical applications may rely on accessible tissues only to assess tissue-specific aging; and forensic settings may rely on accurate age prediction from a variety of tissue sources.

To assess differences across tissues with respect to biological age predictions, we used DNAm data from the Genotype-Tissue Expression (GTEx) Project to obtain epigenetic age estimates for DNAm clocks (Horvath, Hannum, PhenoAge, EpiTOC, EpiClock, AltumAge, Zhang, and Dunedin PACE) across 9 different tissue types (lung, colon, prostate, ovary, breast, kidney, testis, skeletal muscle, and whole blood). We characterize DNAm aging estimates across tissues in terms of the (1) distribution of DNAm clock estimates, (2) strength of each clock’s association with age, (3) correlation of clock estimates between tissue types, and (4) associations of participant characteristics with clock estimates.

## MATERIALS AND METHODS

### Tissue samples

The GTEx Project is a publicly available biobank of >17,000 human tissue samples collected from ~950 post-mortem multi-tissue donors, with ~50 unique human tissue types represented [23]. The GTEx project has generated genome-wide data on genetic variation (using whole-genome sequencing) and gene expression (using RNA sequencing) for >15,000 tissue samples from >800 donors [24]. For each donor, characteristics such as age, race, BMI, and sex are also publicly available [25].

The “enhancing GTEx” (eGTEx) initiative built upon the core GTEx data by adding complementary layers of biological information, including proteomics, DNA and RNA modifications, and telomere length (TL) [26]. As a part of eGTEx, we generated array-based DNAm measurements for ~1,000 GTEx samples, described previously [27]. The selection of tissue types was based on several considerations, including inclusion of cancer-relevant tissues (lung, colon, prostate, ovary, breast, kidney), tissues with unique aging biology (testis, skeletal muscle), and tissues commonly used in epidemiological research (whole blood).

### Genome-wide DNA methylation measurement and processing

We measured DNAm at 866,895 CpG sites throughout the epigenome in 1,000 GTEx tissue samples from 9 unique tissue types obtained from 424 GTEx subjects using the Infinium MethylationEPIC v1 array (Illumina, San Diego, CA, USA). DNA samples were extracted from GTEx tissue samples using Qiagen Gentra Puregene method at GTEx Laboratory Data, Analysis and Coordinating Center (LDACC), and sent to the Institute for Population and Precision Health Laboratory at the University of Chicago on 96-well plates. Neither tissue types nor individuals were not batched by plate. Bisulfite conversion was applied to 500 ng of DNA using EZ-96 DNA methylation kit (Zymo Research, Irvine, CA, USA). All samples were then prepared and analyzed in accordance with the manufacturer guidelines and protocol for the EPIC array.

DNAm data was processed with ChAMP software [28]. Raw DNAm values were background-adjusted using the single sample normal-exponential out-of-band (*ssnoob*) method with dye bias correction [29, 30]. DNAm beta values were normalized using the beta mixture quantile (BMIQ) method, adjusting for type I/II probe bias [31]. After normalization, we removed one additional sample with array-derived genotype profile not matching WGS-derived one. As described previously [27], principal component analysis (PCA) was conducted on DNAm beta values within each tissue type, and 3 samples were removed for being outliers with respect to the top 5 principal components (PCs) of the corresponding tissue. We additionally removed 13 breast samples obtained from males (leaving only female breast samples for analysis), resulting in 973 samples for analyses purposes (representing 9 tissue types and 424 donors). Due to missing telomere and smoking status data, an additional 99 samples were removed (from the regression analyses only) resulting in 874 samples.

### DNAm clock estimates

For this study, we selected several commonly-used clocks, as well as recently developed clocks, including both pan-tissue and blood-specific clocks (Table 1). We excluded clocks that use chronological age as input (i.e., GrimAge). We acknowledge there are additional clocks described in the literature that we are not assessing in this work. DNAm clock estimates for all 973 GTEx tissues samples were obtained using the Horvath group’s online epigenetic clock calculator [32] and the R code provided [33]. Three of the DNAm clocks we analyzed were provided by the calculator: Horvath, Hannum, PhenoAge. Clock estimates for EpiTOC [16], EpiClock [17], AltumAge [34], Zhang clock [19], and DundinPACE [20] were obtained using code provided by the authors. For each of the clock algorithms examined, we provide information on the training data, arrays and CpGs used, the clock’s intended purpose and the number of clock CpGs that were missing from our dataset (i.e., removed during QC) (Table 1).

**Table 1:** Summary of the clock algorithms examined in this work.

**Clock**	**Tissue type(s) for training**	**Array(s) used**	**Purpose**	**Training data age**	**Ref**	**# CpGs used**	**# CpGs missing**
Horvath	Multi-tissue (~8,000 samples)	27K and 450K	Estimate chronological age	Mean: 43 y Range: 0-100 y	33	353	23
Hannum	Whole blood (656 samples)	450K	Estimate chronological age	Mean: ~65 y Range: 19-101y	35	71	9
Pheno Age	Whole blood (9,926 samples)	27K, 450K, and 850K	Health + lifespan estimation	Mean age: 49y Range: 8-80y (NHANES)	15	513	12
Altum Age	Multi-tissue	27K, 450K, and 850K	Estimate chronological age	Mean: ~50 y Range: 0 to >100 y	18	20,318	522
EpiTOC	Fetal tissues and blood	450K	Capture stem cell divisions	Mean: ~65 y Range: 19-101y	16	385	31
EpiClock	Multi-tissue (3,114 samples)	450K, and 850K	Estimate chronological age	Median: 36.5 y Range: 0-103 y	17	6,761	515
Dunedin PACE	Whole blood (1,037 samples)	805K	Estimate pace of aging	Longitudinal birth cohort (sampled at 26, 32, 38, 45 y)	20	173	0
Zhang	Blood/saliva (13,661 samples)	450K and 850K	Estimate chronological age	Mean: 15-82 y (14 cohorts) Range: 2–104 y	19	514	8

The Horvath clock [33] and Hannum clock [35], considered first-generation clocks, were trained with the goal of accurately estimating chronological age based on DNAm values measured from samples. PhenoAge is considered a second-generation clock that reflects both lifespan and health and was trained on age and 9 blood biomarkers [15]. Because the GrimAge clock [14] uses age as an input, we did not evaluate GrimAge in this work, as age prediction is a primary focus of this paper.

EpiTOC (Epigenetic timer of cancer) [16] is a “mitotic” clock was developed to capture the amount of stem cell division in a tissue. EpiTOC uses 385 CpGs selected based on (1) location within promoters that localize to Polycomb group target genes, (2) lack of methylation in a variety of fetal tissue types, and (3) increasing methylation with age in human blood samples.

The EpiClock algorithm was developed to predict chronological age across multiple tissue types [17]. The AltumAge algorithm, another multi-tissue clock, was trained using a neural network approach and is reported to have enhanced performance for older ages and for diverse tissue types.

The Zhang clock was developed from the stated goal of examining whether the association between the age acceleration residual of different predictors (developed by the researchers in the same paper) and death is affected by improving predictive power as its training set sample size increases and by correcting for confounders. The final CpG count was 514, chosen based on their associations with chronological age in blood samples.

DunedinPACE uses DNA methylation data from blood samples for 173 CpGs to determine the “Pace of Aging” of a person. This clock is based on biological changes observed for 19 indicators of organ-system integrity over two decades in the Dunedin birth cohort [20].

Among the CpGs in our post-QC dataset, we were missing a small percentage for the CpGs used by each clock (Table 1).

### Estimation of immune cell infiltration

The presence of leukocytes in GTEx samples was estimated using the LUMP algorithm [36], which leverages data on 44 CpG sites that are specifically methylated specifically in immune cells. LUMP provides a percentage representing “purity”, so we subtracted this percentage from 1 to obtain a percentage represented immune cell infiltration (ICI). To determine if ICI impacts clock performance, we first examined the association between the average immune cell infiltration (for a specific tissue) and the correlation between chronological age and clock age (for a specific tissue), across all eight non-blood tissue types. We also examined the interaction between chronological age and ICI (in relation to clock age) for each clock, in each tissue type.

### Statistical analyses

We examined the distributions of all five biological clock estimates (across tissue types) using histograms, ridge plots, and violin plots. Acceleration for each clock was calculated as the residuals from a linear regression of the clock estimates on chronological age. To examine the strength of associations of clock estimates with participant characteristics (and age), we used linear regression and/or Pearson’s correlation analysis. We calculated (1) the mean deviation for each clock applied to each tissue (i.e., average difference between clock age and chronological age) as well as (2) the median absolute error (i.e., median absolute difference between DNAm age and chronological age), similar to previous work [33]. We also used Pearson’s correlation and linear regression to examine the association between clock estimates obtained from different tissue types. Linear regression models included sex, smoking, BMI, and telomere length as covariates. Telomere length was measured for GTEx samples as previously described [37].

## RESULTS

The characteristics of the GTEx donors for each tissue type used for this project are described in Table 2. The sample sizes for each tissue type ranged from 38 (female breast) to 212 (lung), with a total of 424 donors contributing tissue samples.

**Table 2:** GTEx donor characteristics for each tissue type.

**Total (n=973)**	**Breast (n=38)**	**Colon (n=224)**	**Kidney (n=50)**	**Lung (n=223)**	**Muscle (n=47)**	**Ovary (n=164)**	**Prostate (n=123)**	**Testis (n=50)**	**Whole Blood (n=54)**
**Age (years)**									
Mean (SD)	50.0 (11.9)	56.1 (11.3)	59.7 (8.26)	55.2 (11.1)	57.1 (10.5)	50.6 (13.7)	54.1 (13.0)	54.2 (12.2)	50.3 (12.9)
Median [Min, Max]	50.5 [21, 70]	59.0 [21, 70]	61.5 [36, 70]	57.0 [22, 70]	60.0 [31, 70]	52.0 [21, 70]	57.0 [20, 70]	56.0 [22, 70]	52.5 [22, 70]
Sex									
Male	0 (0%)	156 (69.6%)	39 (78.0%)	160 (71.7%)	28 (59.6%)	0 (0%)	123 (100%)	50 (100%)	45 (83.3%)
Female	38 (100%)	68 (30.4%)	11 (22.0%)	63 (28.3%)	19 (40.4%)	164 (100%)	0 (0%)	0 (0%)	9 (16.7%)
**Race**									
Non-Hispanic White	0 (0%)	3 (1.3%)	0 (0%)	3 (1.3%)	0 (0%)	2 (1.2%)	0 (0%)	0 (0%)	0 (0%)
African American	6 (15.8%)	22 (9.8%)	6 (12.0%)	28 (12.6%)	6 (12.8%)	27 (16.5%)	10 (8.1%)	3 (6.0%)	5 (9.3%)
Other	32 (84.2%)	197 (87.9%)	44 (88.0%)	190 (85.2%)	41 (87.2%)	134 (81.7%)	111 (90.2%)	47 (94.0%)	48 (88.9%)
Missing	0 (0%)	2 (0.9%)	0 (0%)	2 (0.9%)	0 (0%)	1 (0.6%)	2 (1.6%)	0 (0%)	1 (1.9%)
**BMI (kg/m2)**									
Mean (SD)	25.4 (3.94)	27.1 (3.94)	26.4 (3.75)	27.6 (3.91)	26.8 (4.38)	26.8 (4.23)	27.1 (3.81)	27.2 (3.82)	27.4 (4.18)
Median [Min, Max]	25.5 [18.9, 33.3]	27.2 [18.8, 35.0]	26.6 [18.8, 34.8]	27.4 [18.6, 35.0]	26.6 [18.6, 34.4]	26.6 [18.5, 34.9]	27.1 [18.8, 34.9]	27.1 [19.0, 34.8]	27.2 [19.8, 35.0]
**Smoker**									
No	11 (28.9%)	57 (25.4%)	10 (20.0%)	62 (27.8%)	12 (25.5%)	57 (34.8%)	38 (30.9%)	12 (24.0%)	12 (22.2%)
Yes	27 (71.1%)	152 (67.9%)	37 (74.0%)	150 (67.3%)	34 (72.3%)	96 (58.5%)	73 (59.3%)	36 (72.0%)	40 (74.1%)
Missing	0 (0%)	15 (6.7%)	3 (6.0%)	11 (4.9%)	1 (2.1%)	11 (6.7%)	12 (9.8%)	2 (4.0%)	2 (3.7%)
**Telomere length**									
Mean (SD)	1.06 (0.314)	1.09 (0.386)	1.01 (0.339)	0.930 (0.228)	1.39 (0.352)	1.29 (0.291)	1.09 (0.294)	1.97 (0.522)	0.836 (0.206)
Median [Min, Max]	1.02 [0.37, 1.96]	1.07 [0.31, 2.59]	0.96 [0.32, 1.88]	0.91 [0.35, 1.72]	1.34 [0.89, 2.41]	1.25 [0.71, 2.24]	1.05 [0.19, 2.16]	1.93 [1.18, 3.45]	0.87 [0.37, 1.20]
Missing	1 (2.6%)	5 (2.2%)	0 (0%)	19 (8.5%)	4 (8.5%)	13 (7.9%)	0 (0%)	4 (8.0%)	1 (1.9%)

**Figure 1:**
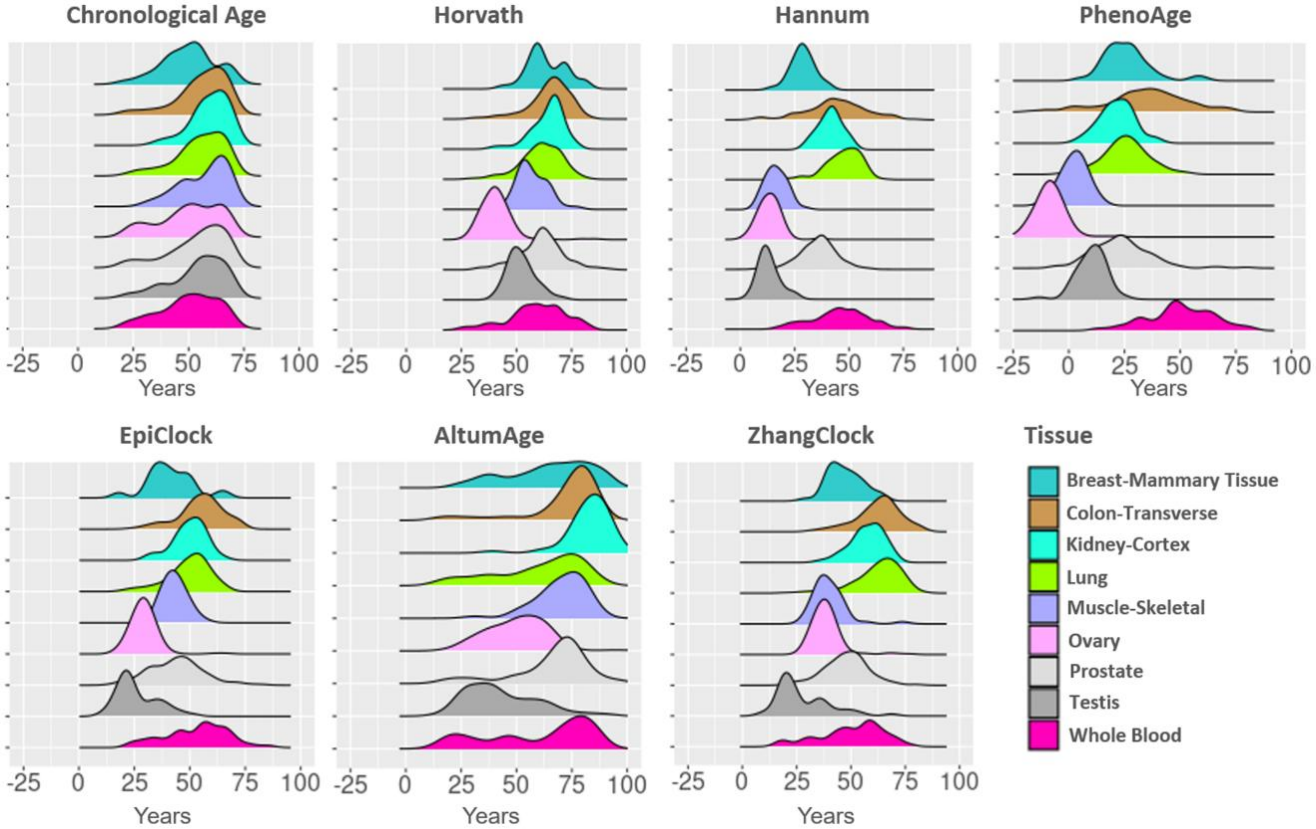
**DNA methylation clock estimates vary across tissue types.** The distributions of estimates for age and six clocks are color coded by tissue type. For each clock, tissue types are ranked by their median and color-coded. The median and inter-quartile ranges are shown as vertical lines.

The tissue-specific distributions of chronological age and estimated DNAm ages (for each clock examined) are shown in Figure 1. The mean chronological age for donors varied somewhat across tissue types (~50 to ~62 years). For each clock examined, estimates varied substantially across tissue types, with mean clock estimates close to 0 years in some scenarios (i.e., PhenoAge for ovary) and close to 100 years other scenarios (i.e., AltumAge for kidney). For each clock, testis and ovary tissue tended to have lower (younger) clock estimates, while colon and lung tended to have higher (older) clock estimates compared to other tissue types.

**Figure 2:**
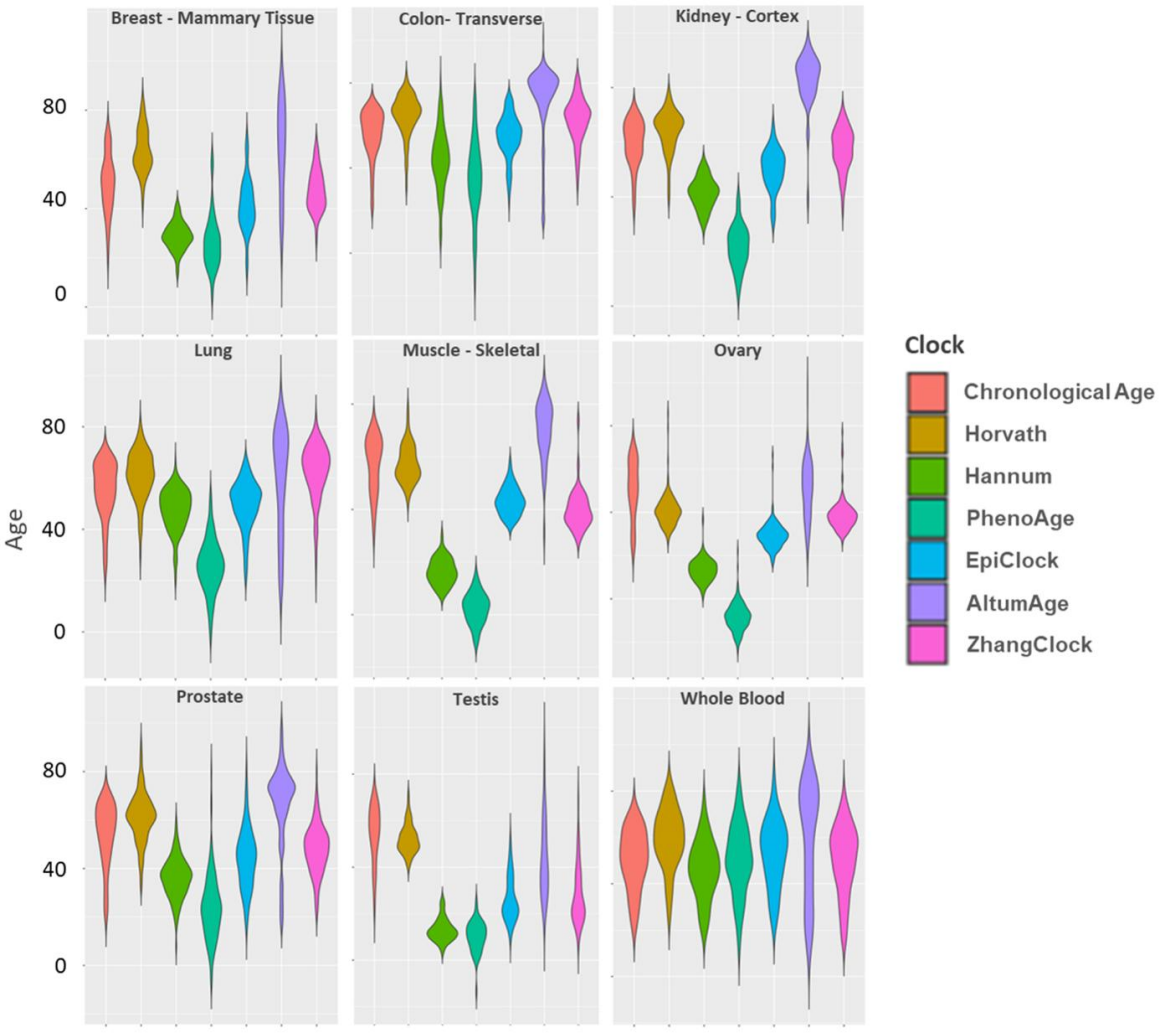
Distributions of chronological age and DNAm clock estimates within each of nine GTEx tissue types.

The distribution of each cock within tissue type is shown in Figure 2. In whole blood, the distribution of each clock (considering both the mean and the variance), was most similar to chronological age, compared to other tissue types. Whole blood had low median absolute errors (Supplementary Table 1) and mean deviations (Supplementary Table 2) compared to other tissue types (for all clocks except AltumAge). The distributions of the Horvath (light brown) and EpiClock (blue) clocks most closely resembled the chronological age distributions (across most tissue types), with correspondingly small median absolute errors and mean deviations (Supplementary Tables 1, 2). The means of the blood-based clocks (PhenoAge and Hannum) tended to be lower than chronological age, with large negative mean deviations, in most tissue types (except in whole blood), and high median absolute errors (Supplementary Tables 1, 2). In general, the clocks’ distributions deviated most from chronological age in muscle, testis, and ovary tissues.

The correlation between each tissue-specific clock estimate and individuals’ chronological age is shown in Table 3. For all clocks except EpiTOC, the clock estimates from blood showed the strongest correlation with chronological age, as compared to all other tissue types. For each clock, the correlation with chronological age varied substantially across tissue types. For most non-blood tissue types (including breast, colon, kidney, lung, ovary, prostate) the Hannum and PhenoAge clocks, both specifically designed for blood DNAm data, showed weaker correlation with chronological age than did the clocks designed for pan-tissue applications (Horvath, AltumAge, and EpiClock). EpiTOC clock was positively correlated with chronological age (P<0.05) in all tissue types except for breast and muscle.

**Table 3:** Peason correlations (and confidence intervals) between clock estimates and chronological age by tissue type.

**Tissue**	**DNAm clocks**						
	**Horvath**	**Hannum**	**PhenoAge**	**EpiClock**	**AltumAge**	**EpiTOC**	**ZhangClock**
Breast	0.78 (0.62-0.88)	0.73 (0.54-0.85)	0.57 (0.30-0.75)	0.86 (0.74-0.92)	0.85 (0.74-0.92)	0.10 (-0.23-0.41)	0.86 (0.74-0.92)
Colon	0.89 (0.86-0.91)	0.39 (0.27-0.50)	0.36 (0.25-0.47)	0.78 (0.72-0.83)	0.88 (0.85-0.91)	0.33 (0.20-0.44)	0.71 (0.63-0.77)
Kidney	0.89 (0.81-0.93)	0.82 (0.69-0.89)	0.67 (0.47-0.80)	0.90 (0.82-0.94)	0.84 (0.73-0.91)	0.49 (0.24-0.68)	0.91 (0.84-0.95)
Lung	0.87 (0.83-0.90)	0.85 (0.81-0.88)	0.71 (0.64-0.77)	0.92 (0.90-0.94)	0.93 (0.91-0.94)	0.36 (0.24-0.47)	0.84 (0.8-0.88)
Muscle	0.54 (0.30-0.72)	0.58 (0.35-0.74)	0.46 (0.19-0.66)	0.72 (0.54-0.83)	0.84 (0.72-0.91)	0.28 (-0.003-0.53)	0.46 (0.2-0.66)
Ovary	0.58 (0.47-0.67)	0.53 (0.41-0.63)	0.26 (0.12-0.40)	0.61 (0.50-0.70)	0.72 (0.64-0.79)	0.16 (0.01-0.31)	0.50 (0.37-0.61)
Prostate	0.86 (0.81-0.90)	0.60 (0.48-0.71)	0.67 (0.56-0.76)	0.81 (0.74-0.86)	0.91 (0.87-0.93)	0.38 (0.22-0.52)	0.71 (0.61-0.79)
Testis	0.69 (0.52-0.82)	0.65 (0.45-0.78)	0.77 (0.62-0.86)	0.52 (0.28-0.70)	0.62 (0.41-0.77)	0.42 (0.16-0.62)	0.45 (0.19-0.65)
Whole Blood	0.92 (0.87-0.96)	0.93 (0.89-0.96)	0.88 (0.81-0.93)	0.95 (0.92-0.97)	0.94 (0.90-0.96)	0.41 (0.16-0.61)	0.94 (0.9-0.97)

**Figure 3:**
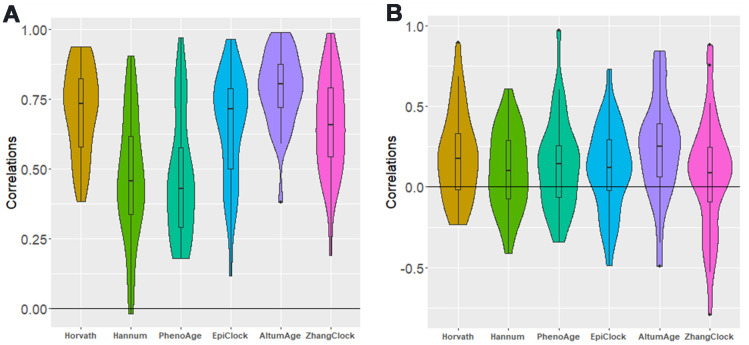
**Distribution of between-tissue correlations for each clock, for all possible pairs of tissue types.** (**A**) not adjusted for age (**B**) adjusted for age. Each of the five distributions shown (in both panels) has a mean greater than zero (P<0.05).

**Figure 4:**
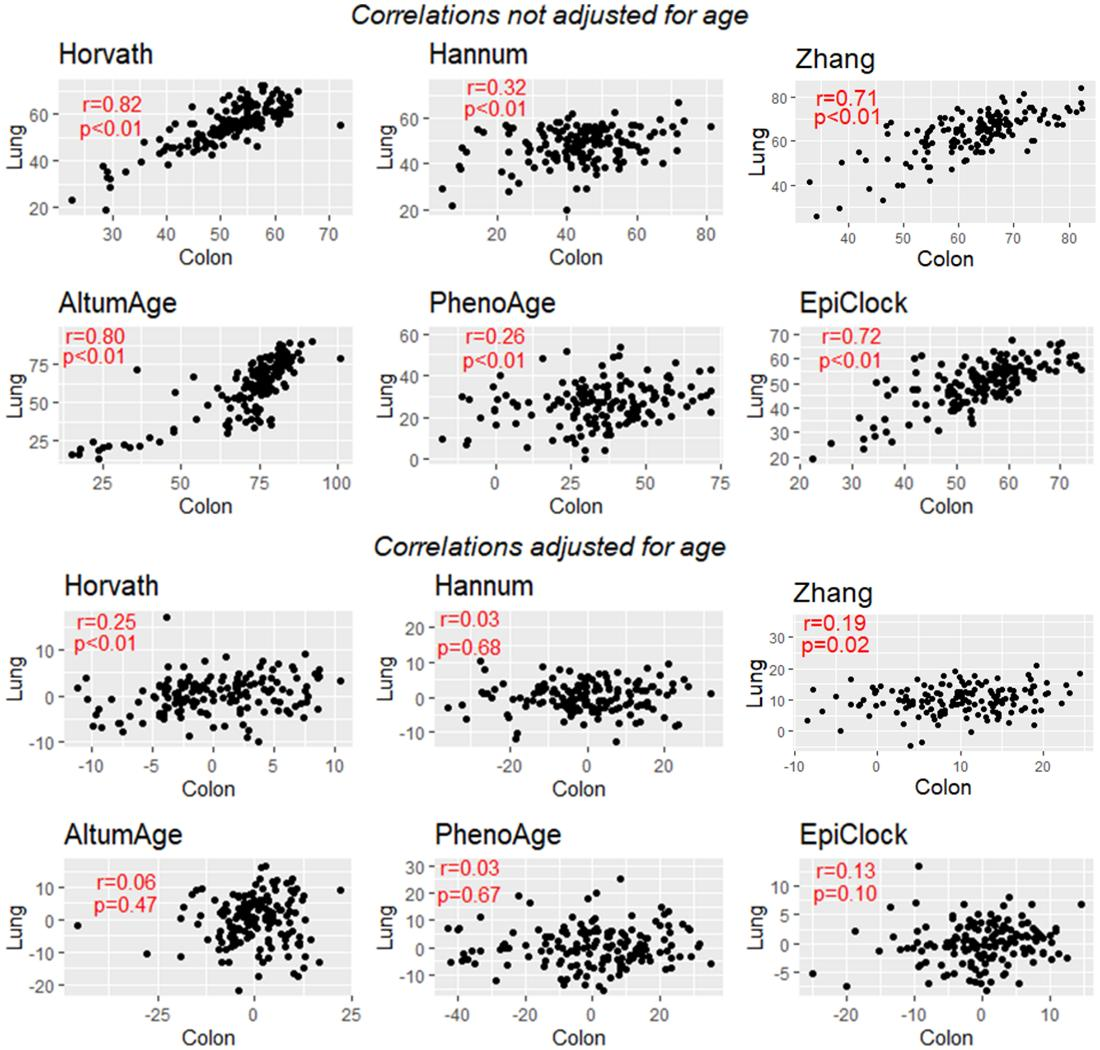
**Correlation between lung-based and colon-based DNAm clock estimates for 6 clocks, without adjustment for age (top) and with adjustment for age (bottom).** EpiTOC showed very weak evidence for correlation and is not presented.

For each clock, we attempted to estimate the correlation in clock estimates between pairs of tissue types, using data from donors with DNAm data available for both tissue types. For each clock, there was clear correlation across tissue types (Supplementary Tables 3–9 and Supplementary Figures 1–6), with an average tissue-tissue correlation of 0.71 for Horvath, 0.47 for Hannum, 0.47 for PhenoAge, 0.15 for EpiTOC, 0.78 for AltumAge, 0.65 for EpiClock, and 0.65 for Zhang (based on 32 possible tissue-tissue pairs) (Figure 3A). Since lung and colon had the largest sample size (146 donors with DNAm data for both tissue types) and the most power/precision for assessing correlation between clock estimates, we present those results in more detail in Figure 4. Specifically, we observed the strongest correlation between lung and colon using Horvath (r=0.82) and AltumAge (r=0.80) (Figure 4, top).

As expected, after adjusting for age, the inter-tissue correlations for each clock were substantially attenuated. Examining age-adjusted associations across all possible pairs of tissue types (Supplementary Tables 3–10 and Supplementary Figures 1–6), we find evidence of weak residual correlation in clock estimates between tissues after age adjustment (Figure 3B). The means of the distributions of age-adjusted correlations for all possible tissue pairs correlations were greater than zero for each clock examined (EpiTOC P=0.003, Hannum P=0.02, Horvath P=0.0003, PhenoAge P=0.01, EpiClock P=0.02, AltumAge P <0.001, Zhang P=0.02). Figure 4 (bottom) shows the correlation between the age-adjusted clock estimates for lung and colon, with only the Horvath clock showing a correlation with P<0.05.

To assess the hypothesis that clock performance varies across tissue types due to differences in immune cell infiltration (ICI), we first examined the average leukocyte percentage in each tissue type. Based on visual inspection, there was suggestive evidence that tissue types with higher leukocyte percentage showed stronger correlation between chronological age and clock age (Supplementary Figure 7). However, given the small number of tissue types (8), we were underpowered for statistical testing, although AltumAge and EpiClock had P-values of 0.10 and 0.11, respectively.

To determine if ICI impacts clock performance, we examined with interaction between chronological age and leukocyte percentage (in relation to clock age) in a linear regression model. For some tissue types with small sample sizes (breast, kidney, muscle), the association between chronological age and clock age was no longer clear after including the interaction (P>0.05 for most clocks), suggesting lack of power for interaction testing in these tissue types (Supplementary Table 11). However, lung showed the clearest evidence that clock performance strengthened as ICI increased, with interaction P<0.05 for Horvath, Hannum, Zhang, EpiClock, PhenoAge, and EpiTOC (Supplementary Table 11).

We examined the association of participant characteristics (sex, BMI, smoking, and TL) with clock estimates for each tissue type (Supplementary Tables 12, 13). Of note, smoking showed association (P<0.05) with increased DNAm clock estimates for the Horvath clock (in testis), the Hannum clock (in lung and testis), the PhenoAge clock (in lung), EpiTOC (in lung), EpiClock (in lung and testis), and AltumAge (in lung and testis) (Table 4). For the smoking analyses, 72 tests were conducted, thus the Bonferroni-corrected P-value threshold is 0.0006. PACE (blood and lung), PhenoAge (lung), and EpiClock (lung) pass this threshold.

**Table 4:** Beta coefficients (and P-values) for the association between smoking and age acceleration, by tissue type and clock.

	**Blood (n=51)**	**Breast (n=36)**	**Colon (n=205)**	**Kidney (n=47)**	**Lung (n=194)**	**Muscle (n=43)**	**Ovary (n=142)**	**Prostate (n=111)**	**Testis (n=45)**
EpiClock	**3.82 (0.026)**	0.02 (0.991)	-1.52 (0.128)	-0.98 (0.454)	**2.00 (<0.001)**	-1.96 (0.104)	0.02 (0.973)	0.12 (0.933)	**5.12 (0.037)**
AltumAge	**6.96 (0.024)**	-5.55 (0.125)	-0.69 (0.557)	-3.49 (0.071)	**3.64 (0.002)**	-2.22 (0.313)	2.02 (0.185)	-1.27 (0.354)	6.82 (0.103)
Horvath	3.76 (0.055)	-0.39 (0.841)	0.13 (0.328)	**-4.13 (0.003)**	-0.39 (0.593)	**-3.34 (0.039)**	1.29 (0.078)	-0.81 (0.438)	**4.52 (0.032)**
Hannum	1.03 (0.565)	-2.28 (0.073)	-3.43 (0.097)	-0.04 (0.738)	**1.96 (0.005)**	-1.83 (0.152)	-0.65 (0.339)	-1.39 (0.234)	1.23 (0.279)
PhenoAge	1.62 (0.557)	-4.20 (0.193)	-3.53 (0.20)	-1.92 (0.369)	**4.92 (<0.001)**	-0.97 (0.596)	0.69 (0.449)	-0.38 (0.838)	1.94 (0.205)
EpiTOC	0.00 (0.838)	-0.01 (0.241)	**-0.01 (0.027)**	-0.00 (0.599)	**0.01 (0.003)**	-0.00 (0.883)	0.00 (0.301)	0.00 (0.979)	0.00 (0.204)
Zhang	-2.32 (0.214)	-1.32 (0.339)	**-2.18 (0.038)**	-0.09 (0.940)	0.02 (0.980)	-3.82 (0.080)	0.01 (0.051)	-0.378 (0.221)	6.45 (0.084)
PACE	**0.009 (<0.001)**	**0.118 (0.003)**	**0.064 (0.014)**	0.042 (0.224)	**0.083 (<0.001)**	0.006 (0.805)	0.007 (0.547)	0.034 (0.222)	-0.008 (0.588)

Linear regression models include sex, BMI, and telomere length (TQI) as covariates. Association estimates with P-vales <0.05 are shown in bold text. Smoking is coded as never (0) and ever (1).

## DISCUSSION

In this study we have applied eight DNAm aging/clock algorithms to DNAm data derived from 9 different human tissue types, and we demonstrate that there are substantial differences in these clock estimates across tissue types. We found that the tissue types examined varied in terms of their mean clock estimates, as well as the strength of the correlation between the clock estimates and chronological age. These differences across tissue types were most apparent for clocks trained using DNAm from blood only (e.g., Hannum), but also present for clocks trained on multiple tissue types (e.g., Horvath, a clock designed for pan-tissue age prediction). When applied to different tissue types, each clock showed strong correlations of epigenetic age across tissues, but these correlations were drastically (but not completely) attenuated after adjustment for age, suggesting age acceleration estimates from a single tissue type (e.g., whole blood) can potentially serve as a proxy for other tissue types, although perhaps a weak proxy. Therefore, studies of additional tissue types are needed.

We acknowledge that some of the clocks examined here (Hannum, PhenoAge), were not developed with the goal of being applied to DNAm data from non-blood tissue types. Therefore, one should not expect these clocks to predict age (or other aging-related phenotypes) equally well across tissue types. The pan-tissue clocks tended to be better predictors of chronological age for non-blood tissues, but prediction for these clocks tended to be worse for muscle, ovary, and testis (when compared to lung, colon, breast, kidney, and prostate). Of note, ovary and testis were not in the Horvath training sample. All clocks showed the strongest correlation with chronological age when applied to DNAm data from blood (including the pan-tissue clocks), which likely reflects the fact that blood DNAm data is the most common type of data in the datasets used to train the clocks examined in this work. Furthermore, we provide suggestive evidence that higher immune cell infiltration into non-blood tissues leads to improved clock performance. We also acknowledge that mitotic clocks, such as EpiTOC, are not designed to predict chronological age, although clear correlation with age is observed across all tissue types.

The clocks varied with respect to their mean estimate across tissues, with AltumAge tending to provide the highest age estimates and PhenoAge tending to provide the lowest age estimates. For all clocks, the mean of the clock estimate tended to be closest to mean for chronological age when applied to blood DNAm data. It is possible that some differences in performance observed among clocks may be due in part to differences in the age distribution of the clock’s training dataset compared to our GTEx sample.

The effects of smoking on DNAm clock estimates appeared more pronounced in lung tissue (and perhaps in blood and testicular tissue) as compared to other tissues, with smoking being generally associated with increased DNAm aging across clocks. This observation likely reflects the fact that lung tissue is exposed to tobacco combustion products directly via inhalation, whereas other tissue types are primarily exposed to tobacco combustion byproducts from the blood stream. The association of smoking with accelerated aging in testicular tissue is novel and requires validation in future studies. No other donor characteristics showed clear evidence of consistent association with clock estimates across tissue types (Supplementary Tables 12, 13).

We observed that EpiTOC estimates generally showed weaker correlations with chronological age compared to the other DNAm clocks examined. EpiTOC differs from other clocks examined here in that it is a “mitotic clock” developed to reflect the number of stem cell divisions that have occurred in a tissue’s corresponding stem cell population over time rather than being trained to predict chronological age [38].

Our results suggest that forensic applications of DNAm clocks using non-blood tissue types will provide age estimates that are not as accurate as predictions based on blood, especially if using clocks algorithms trained on blood samples. Our results also suggest that tissue-specific DNAm aging measures may have utility for detecting biological differences in organ aging, as accelerated DNAm aging due to smoking was more pronounced in lung compared to other tissue types. However, in order to draw robust conclusions regarding the consistency of association between participant characteristics and DNAm clocks across tissue types, larger samples sizes are needed.

While our study comprehensively examines how chronological age associates with epigenetic age across different tissue types using 8 DNAm clocks, there were several limitations. First, our study included some tissue types with small sample sizes (~50 samples), which limited our power to detect associations between epigenetic age and donor characteristics. Larger studies of DNAm across multiple tissue/cell types (with more metadata on donors) are needed to better understand how DNAm clocks (and individual CpG sites) relate to donor characteristics. Second, we have a limited understanding of the cell type heterogeneity of bulk tissue samples from which our DNAm data is derived. This heterogeneity may influence the performance of the clock algorithms we are studying, as we demonstrate by examining the impact of leukocyte infiltration on clock performance. Cell type deconvolution approaches have been shown to be useful for adapting blood-based clocks to perform well in other tissues, such as saliva [39]. Third, the GTEx cohort of tissue donors, all of whom were selected for inclusion in GTEx post-mortem, is strongly enriched for smokers and individuals dying at younger ages; this potential selection bias may introduce bias into the associations between epigenetic and chronological age reported in this study. Furthermore, while our study examines DNAm clocks in nine tissue types, future studies that include additional tissue types will improve our understanding of how epigenetic aging varies across the human body.

## Supplementary Material

Supplementary Figures

Supplementary Tables 1-10

Supplementary Table 11

Supplementary Tables 12-13
