# Supplementary material for: Characterization of DNA methylation clock algorithms applied to diverse tissue types
Source: Aging (Albany NY). 2025 Jan 3;17(1):67–96. doi: 10.18632/aging.206182 (PMC11810061; doi:10.18632/aging.206182)
Supplement: Supplementary Figures [file aging-17-206182-s001.pdf]

SUPPLEMENTARY FIGURES

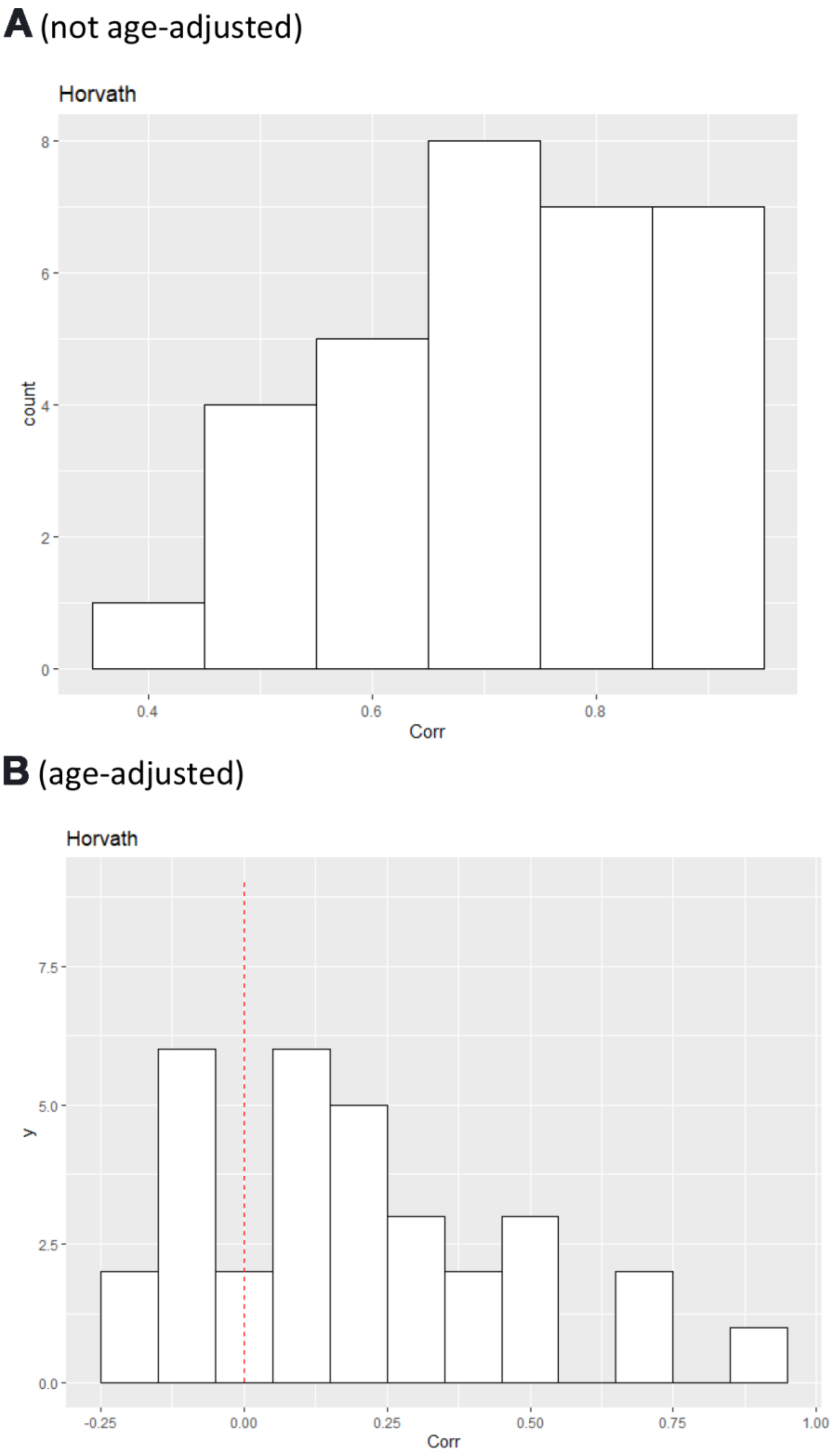

Supplementary Figure 1. Distribution of Correlation Estimates (from Supplementary Table 3) for all Tissue pairs for the Horvath Clock (**A**), not age-adjusted; (**B**) age-adjusted.

A(not age adjusted)

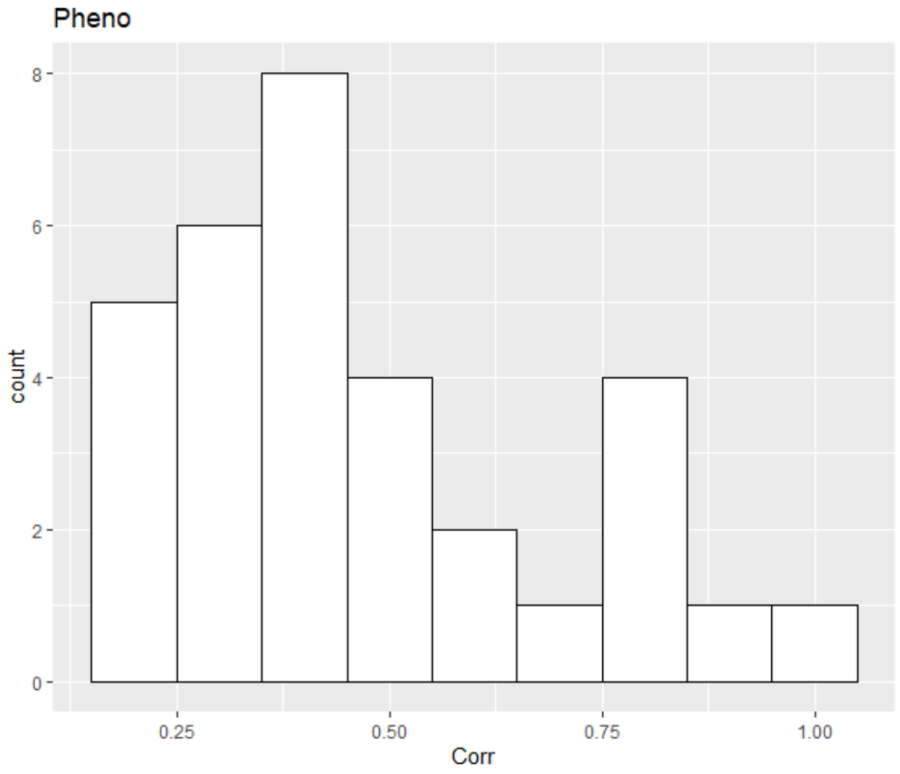

B(age-adjusted)

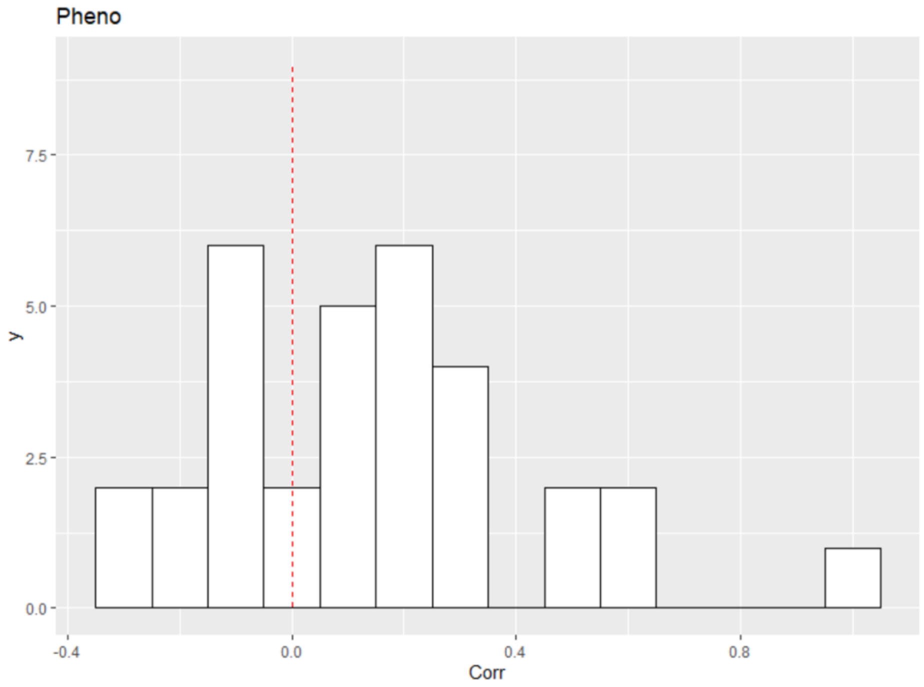

Supplementary Figure 2. Distribution of Correlation Estimates (from Supplementary Table 4) for all Tissue pairs for the PhenoAge Clock (**A**), not age-adjusted; (**B**) age-adjusted.

A (not age-adjusted)

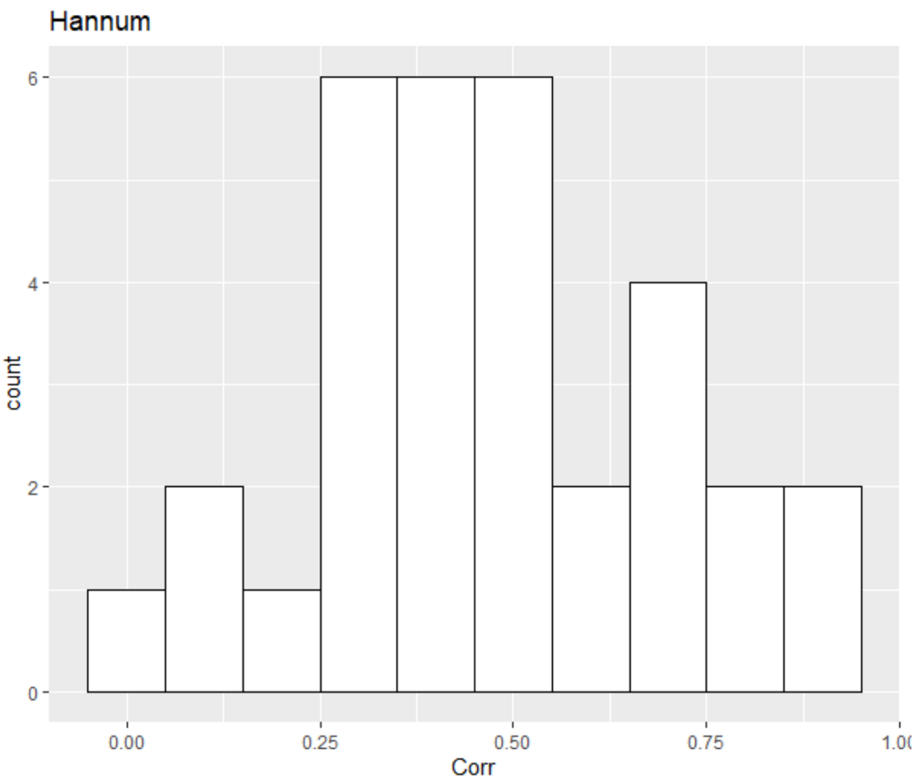

B (age adjusted)

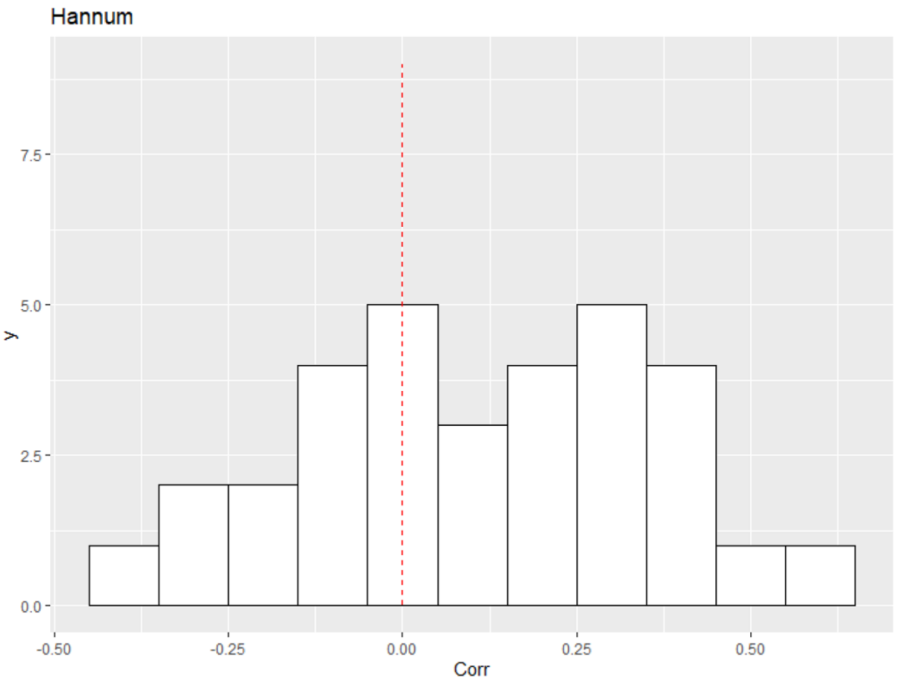

Supplementary Figure 3. Distribution of Correlation Estimates (from Supplementary Table 5) for all Tissue pairs for the Hannum Clock (**A**), not age-adjusted; (**B**) age-adjusted.

A (not age-adjusted)

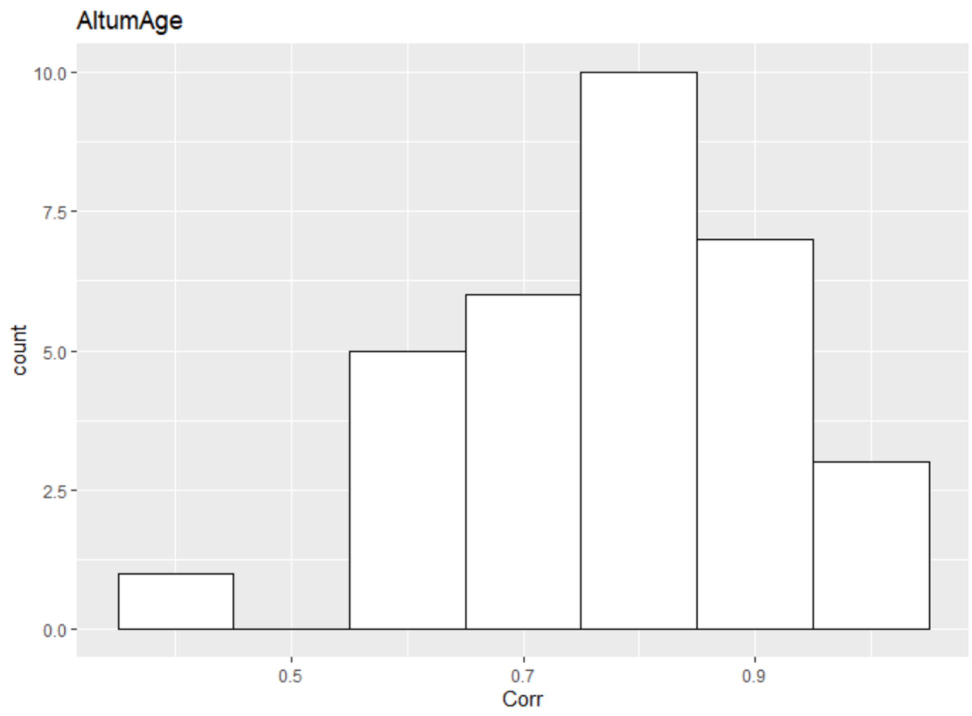

B (age-adjusted)

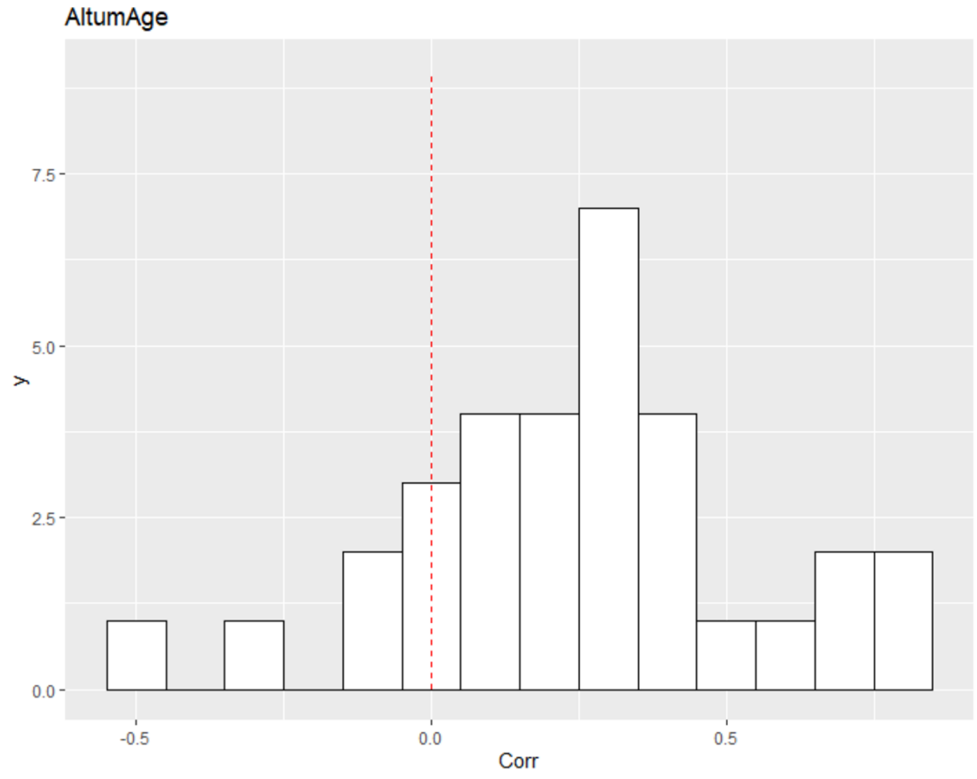

Supplementary Figure 4. Distribution of Correlation Estimates (from Supplementary Table 6) for all Tissue pairs for the AltumAge Clock (**A**), not age-adjusted; (**B**) age-adjusted.

A (not age-adjusted)

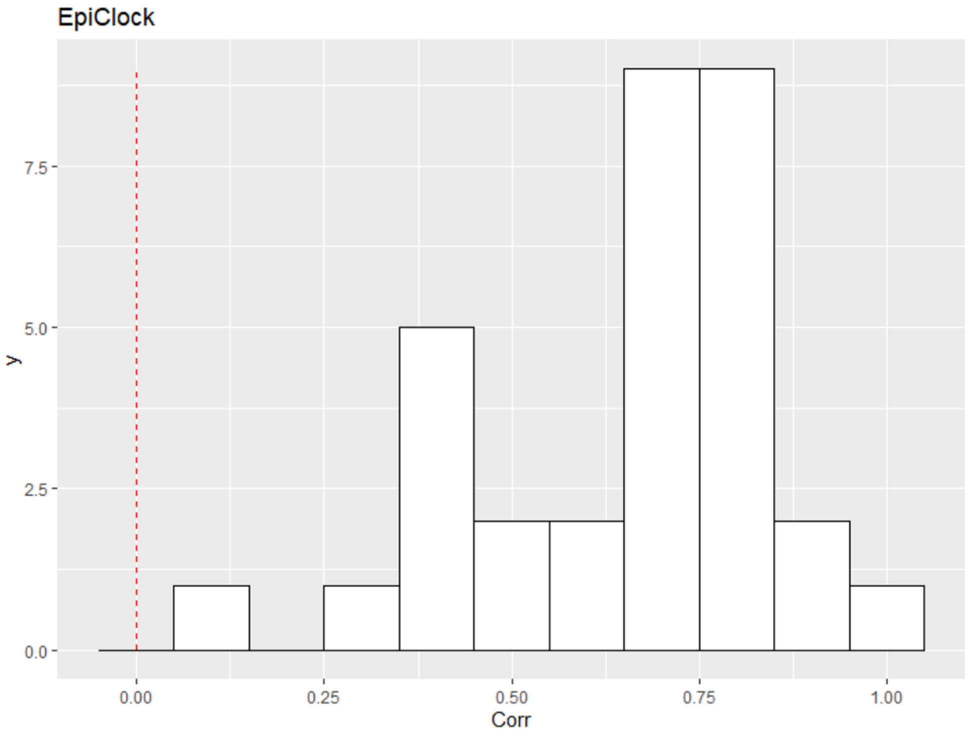

B (age-adjusted)

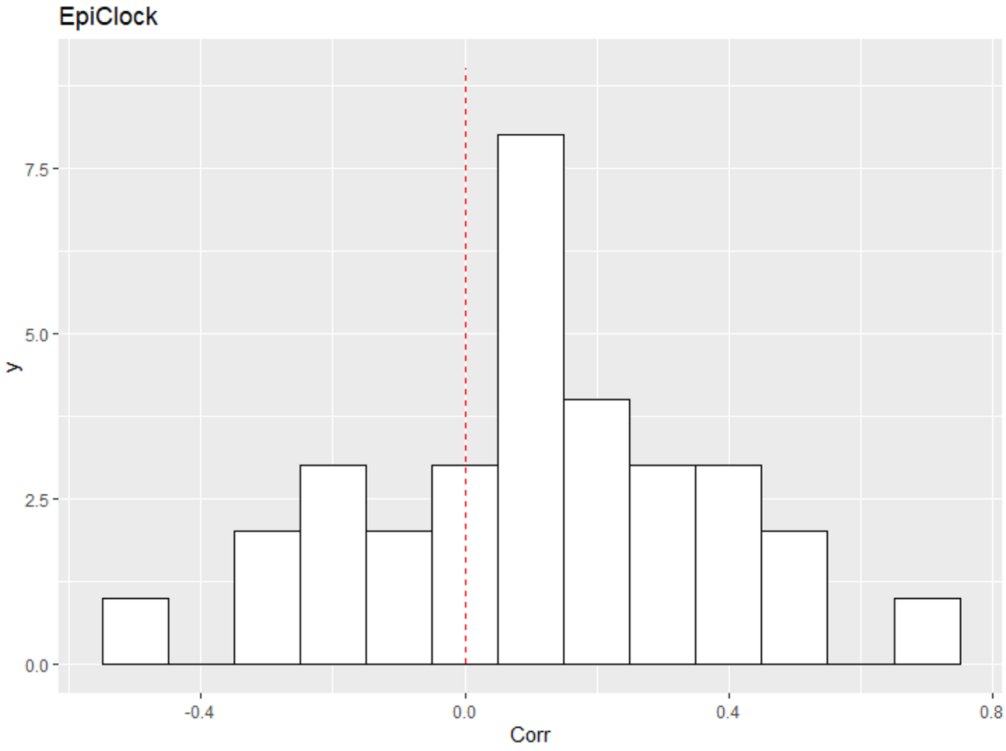

Supplementary Figure 5. Distribution of Correlation Estimates (from Supplementary Table 7) for all Tissue pairs for EpiClock (**A**), not age-adjusted; (**B**) age-adjusted.

A (not age-adjusted)

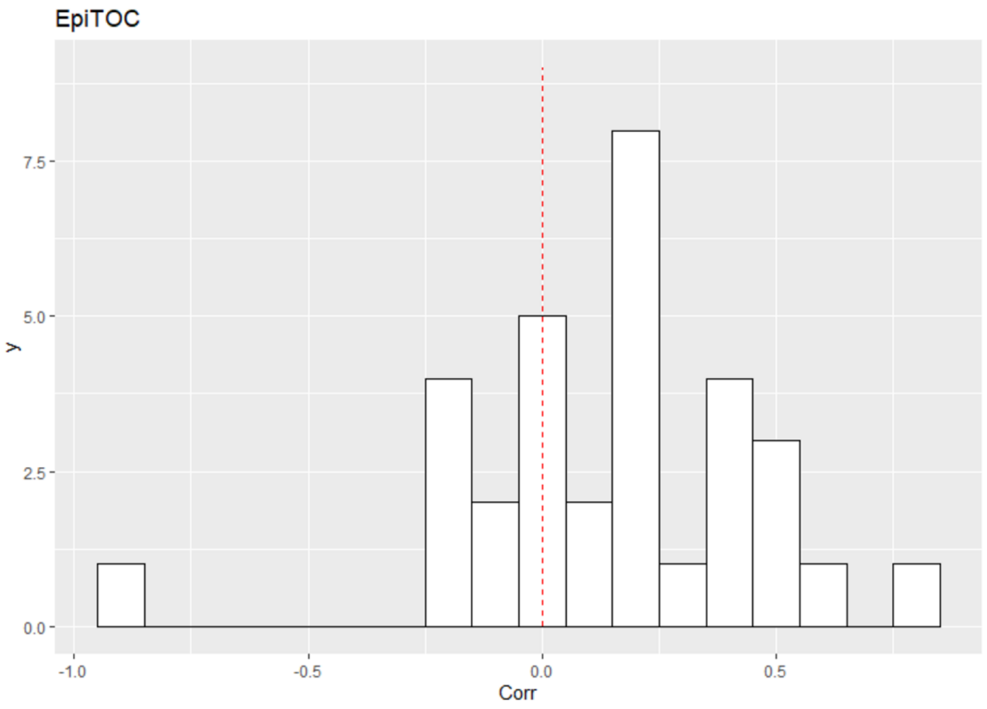

B (age-adjusted)

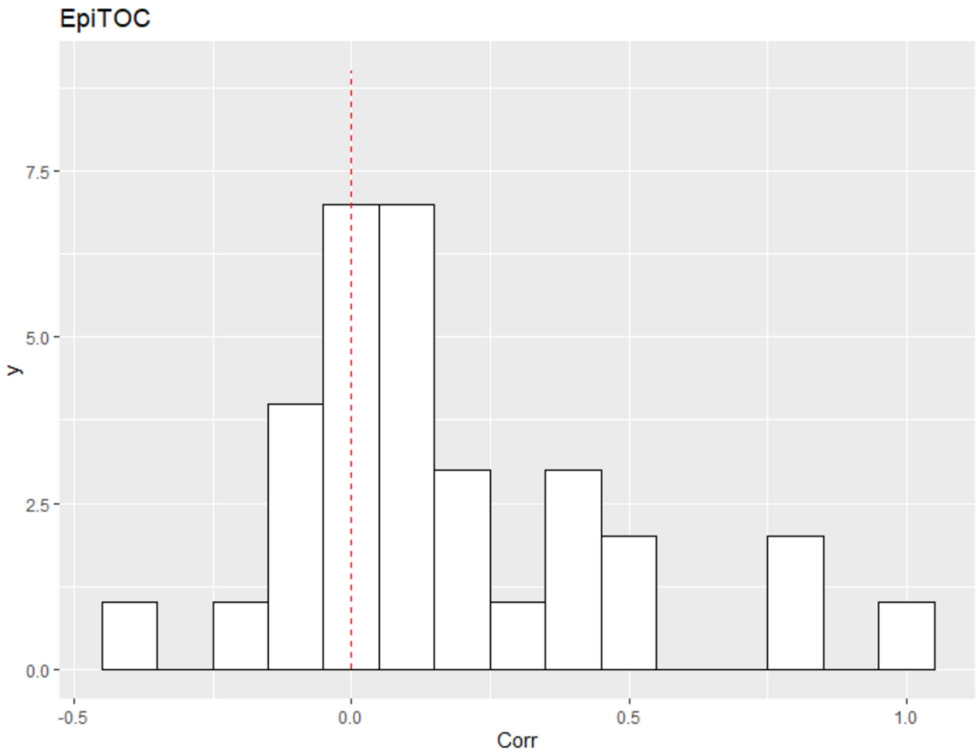

Supplementary Figure 6. Distribution of Correlation Estimates (from Supplementary Table 8) for all Tissue pairs for EpiTOC (**A**), not age-adjusted; (**B**) age-adjusted.

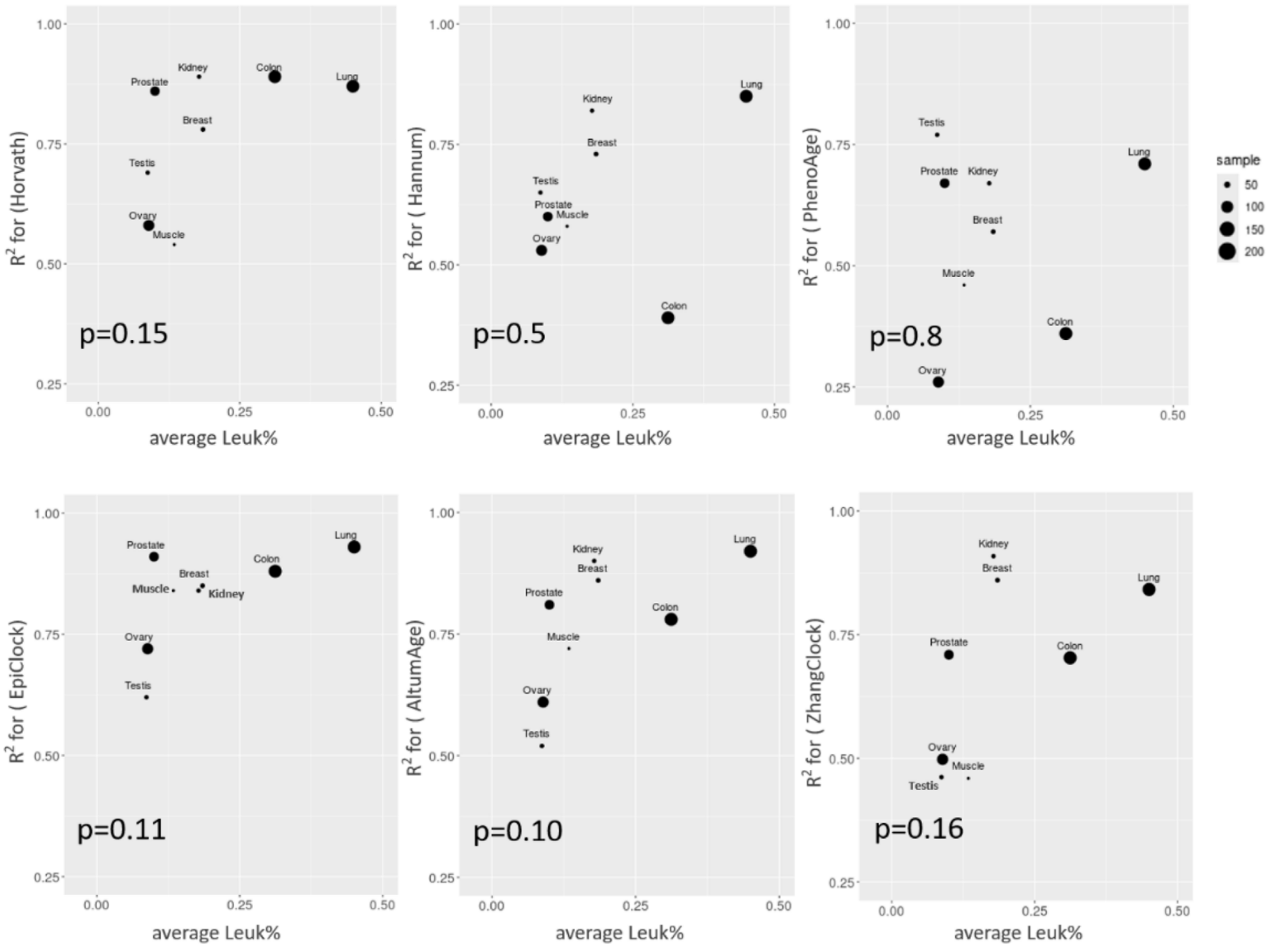

Supplementary Figure 7. Scatter plot of average leukocyte percentage (from LUMP) vs. the R^2 for each clock's association with chronological age. P-values correspond to the association between leukocyte percentage and the R^2 value (n=8).
