# Supplementary material for: Characterization of DNA methylation clock algorithms applied to diverse tissue types
Source: Aging (Albany NY). 2025 Jan 3;17(1):67–96. doi: 10.18632/aging.206182 (PMC11810061; doi:10.18632/aging.206182)
Supplement: Supplementary Tables 1-10 [file aging-17-206182-s002.pdf]

SUPPLEMENTARY TABLES

Supplementary Table 1. Median absolute error/difference for each clock, by tissue type.

Clock	Breast	Colon	Kidney	Lung	Muscle	Ovary	Prostate	Testis	Blood
AltumAge	16.17	17.72	22.49	10.51	14.17	6.333	12.65	14.26	13.51
Horvath	3.524	4.974	3.375	3.991	14.72	25.56	4.732	20.38	2.976
PhenoAge	23.63	21.26	38.27	29.98	55.50	60.87	30.40	43.86	4.807
EpiClock	8.644	4.564	9.766	5.655	15.93	23.07	10.89	29.12	2.607
Hannum	22.07	13.66	18.44	7.733	41.20	39.54	20.44	43.08	5.198
Zhang	4.534	7.177	2.781	7.814	18.43	14.57	7.647	29.36	2.016

Supplementary Table 2. Average age acceleration (mean deviation) for each clock, by tissue type.

Clock	Blood	Breast	Colon	Kidney	Lung	Muscle	Ovary	Prostate	Testes
Horvath	-1.56	0.85	-4.75	-2.63	-0.20	-13.45	-23.74	0.05	-18.58
Altum	9.12	14.89	15.94	23.11	4.73	13.16	-0.36	12.09	-10.82
EpiClock	2.01	-8.65	-1.07	-9.74	-5.57	-14.01	-21.44	-10.23	-28.06
Hannum	-4.38	-21.29	-11.90	-18.33	-7.63	-40.77	-37.34	-18.17	-41.21
PhenoAge	-0.17	-23.80	-21.32	-38.07	-29.67	-54.79	-59.12	-30.42	-44.06
Zhang	0.22	-2.96	7.24	-2.10	7.70	-18.17	-12.79	-6.26	-29.20

Supplementary Table 3. Correlation between tissue types for the Horvath clock.

Non-adjusted correlation	Age-adjusted correlation	Tissue type pair	Sample size	Chronological age range
0.769	0.249	Breast Colon	15	21-70
0.927	-0.115	Breast Kidney	5	42-66
0.656	0.231	Breast Lung	16	37-70
0.938	0.894	Breast Muscle	4	40-66
0.809	0.248	Breast Ovary	28	21-69
0.871	-0.065	Breast Blood	5	42-66
0.731	0.211	Colon Kidney	38	36-70
0.819	0.254	Colon Lung	153	22-70
0.573	-0.051	Colon Muscle	28	31-70
0.457	-0.065	Colon Ovary	53	21-70
0.842	0.223	Colon Prostate	85	22-70
0.682	0.111	Colon Testis	38	22-70
0.924	0.532	Colon Blood	45	22-70
0.724	0.438	Kidney Lung	26	39-68
0.893	0.684	Kidney Muscle	12	42-70
0.738	0.656	Kidney Ovary	11	42-68
0.771	0.303	Kidney Prostate	30	36-70
0.384	-0.234	Kidney Testis	27	36-70
0.721	0.101	Kidney Blood	24	36-70
0.557	0.111	Lung Muscle	25	31-67
0.469	-0.125	Lung Ovary	51	22-70
0.789	0.132	Lung Prostate	73	22-70
0.476	0.058	Lung Testis	33	25-68
0.897	0.546	Lung Blood	37	22-68
0.635	0.377	Muscle Ovary	12	34-70
0.761	0.461	Muscle Prostate	16	31-70
0.475	0.028	Muscle Testis	11	31-70
0.742	-0.004	Muscle Blood	11	31-70
0.581	-0.148	Ovary Blood	9	42-66
0.576	-0.201	Prostate Testis	39	22-70
0.893	0.317	Prostate Blood	33	22-70
0.671	0.145	Testis Blood	27	22-70

Supplementary Table 4. Correlation between tissue types for the PhenoAge clock.

Non-adjusted correlation	Age-adjusted correlation	Tissue type pair	Sample size	Chronological age range
0.483	0.338	Breast Colon	15	21-70
0.852	0.556	Breast Kidney	5	42-66
0.179	-0.341	Breast Lung	16	37-70
0.971	0.971	Breast Muscle	4	40-66
0.305	0.144	Breast Ovary	28	21-69
0.435	-0.206	Breast Blood	5	42-66
0.411	0.072	Colon Kidney	38	36-70
0.259	0.035	Colon Lung	153	22-70
0.299	0.241	Colon Muscle	28	31-70
0.221	0.185	Colon Ovary	53	21-70
0.266	-0.046	Colon Prostate	85	22-70
0.328	-0.149	Colon Testis	38	22-70
0.479	0.189	Colon Blood	45	22-70
0.185	-0.197	Kidney Lung	26	39-68
0.424	-0.126	Kidney Muscle	12	42-70
0.231	-0.281	Kidney Ovary	11	42-68
0.448	0.142	Kidney Prostate	30	36-70
0.423	-0.099	Kidney Testis	27	36-70
0.522	-0.056	Kidney Blood	24	36-70
0.426	0.151	Lung Muscle	25	31-67
0.218	0.056	Lung Ovary	51	22-70
0.415	-0.085	Lung Prostate	73	22-70
0.563	0.219	Lung Testis	33	25-68
0.777	0.476	Lung Blood	37	22-68
0.271	0.261	Muscle Ovary	12	34-70
0.449	0.243	Muscle Prostate	16	31-70
0.791	0.341	Muscle Testis	11	31-70
0.801	0.474	Muscle Blood	11	31-70
0.615	0.601	Ovary Blood	9	42-66
0.533	-0.058	Prostate Testis	39	22-70
0.724	0.069	Prostate Blood	33	22-70
0.801	0.251	Testis Blood	27	22-70

Supplementary Table 5. Correlation between tissue types for the Hannum clock.

Non-adjusted correlation	Age-adjusted correlation	Tissue type pair	Sample size	Chronological age range
0.417	0.152	Breast Colon	15	21-70
0.881	0.606	Breast Kidney	5	42-66
0.489	-0.084	Breast Lung	16	37-70
0.066	0.338	Breast Muscle	4	40-66
0.479	0.271	Breast Ovary	28	21-69
0.654	0.183	Breast Blood	5	42-66
0.729	0.439	Colon Kidney	38	36-70
0.318	0.034	Colon Lung	153	22-70
0.433	0.391	Colon Muscle	28	31-70
0.109	-0.014	Colon Ovary	53	21-70
0.381	0.095	Colon Prostate	85	22-70
0.266	-0.181	Colon Testis	38	22-70
0.532	0.274	Colon Blood	45	22-70
0.493	0.017	Kidney Lung	26	39-68
0.541	-0.071	Kidney Muscle	12	42-70
0.337	-0.159	Kidney Ovary	11	42-68
0.414	-0.041	Kidney Prostate	30	36-70
0.422	-0.042	Kidney Testis	27	36-70
0.743	0.108	Kidney Blood	24	36-70
0.606	0.327	Lung Muscle	25	31-67
0.336	-0.251	Lung Ovary	51	22-70
0.579	0.0899	Lung Prostate	73	22-70
0.391	-0.129	Lung Testis	33	25-68
0.905	0.462	Lung Blood	37	22-68
0.191	0.178	Muscle Ovary	12	34-70
0.512	0.391	Muscle Prostate	16	31-70
0.337	-0.109	Muscle Testis	11	31-70
0.695	0.153	Muscle Blood	11	31-70
-0.018	-0.412	Ovary Blood	9	42-66
0.314	-0.268	Prostate Testis	39	22-70
0.766	0.261	Prostate Blood	33	22-70
0.767	0.396	Testis Blood	27	22-70

Supplementary Table 6. Correlation between tissue types for the AltumAge clock.

Non-adjusted correlation	Age-adjusted correlation	Tissue type pair	Sample size	Chronological age range
0.774	0.252	Breast Colon	15	21-70
0.991	0.711	Breast Kidney	5	42-66
0.711	0.074	Breast Lung	16	37-70
0.957	0.844	Breast Muscle	4	40-66
0.724	0.222	Breast Ovary	28	21-69
0.868	-0.493	Breast Blood	5	42-66
0.861	0.518	Colon Kidney	38	36-70
0.792	0.059	Colon Lung	153	22-70
0.811	0.347	Colon Muscle	28	31-70
0.591	0.064	Colon Ovary	53	21-70
0.896	0.343	Colon Prostate	85	22-70
0.608	0.035	Colon Testis	38	22-70
0.963	0.395	Colon Blood	45	22-70
0.752	0.383	Kidney Lung	26	39-68
0.935	0.841	Kidney Muscle	12	42-70
0.728	0.393	Kidney Ovary	11	42-68
0.811	0.266	Kidney Prostate	30	36-70
0.382	-0.111	Kidney Testis	27	36-70
0.837	0.201	Kidney Blood	24	36-70
0.804	0.167	Lung Muscle	25	31-67
0.676	-0.141	Lung Ovary	51	22-70
0.831	-0.009	Lung Prostate	73	22-70
0.647	0.324	Lung Testis	33	25-68
0.949	0.639	Lung Blood	37	22-68
0.929	0.703	Muscle Ovary	12	34-70
0.848	0.181	Muscle Prostate	16	31-70
0.648	0.251	Muscle Testis	11	31-70
0.806	0.397	Muscle Blood	11	31-70
0.743	-0.344	Ovary Blood	9	42-66
0.632	-0.042	Prostate Testis	39	22-70
0.908	0.062	Prostate Blood	33	22-70
0.749	0.319	Testis Blood	27	22-70

Supplementary Table 7. Correlation between tissue types for Epiclock.

Non-adjusted correlation	Age-adjusted correlation	Tissue type pair	Sample size	Chronological age range
0.834	0.414	Breast Colon	15	21-70
0.836	-0.221	Breast Kidney	5	42-66
0.758	-0.084	Breast Lung	16	37-70
0.786	0.423	Breast Muscle	4	40-66
0.725	0.308	Breast Ovary	28	21-69
0.772	-0.485	Breast Blood	5	42-66
0.751	0.141	Colon Kidney	38	36-70
0.717	0.131	Colon Lung	153	22-70
0.555	0.235	Colon Muscle	28	31-70
0.329	-0.152	Colon Ovary	53	21-70
0.713	0.122	Colon Prostate	85	22-70
0.507	0.069	Colon Testis	38	22-70
0.857	0.093	Colon Blood	45	22-70
0.799	0.467	Kidney Lung	26	39-68
0.681	0.315	Kidney Muscle	12	42-70
0.378	-0.286	Kidney Ovary	11	42-68
0.659	0.211	Kidney Prostate	30	36-70
0.392	0.064	Kidney Testis	27	36-70
0.891	0.457	Kidney Blood	24	36-70
0.746	0.213	Lung Muscle	25	31-67
0.449	-0.301	Lung Ovary	51	22-70
0.732	0.044	Lung Prostate	73	22-70
0.367	-0.129	Lung Testis	33	25-68
0.964	0.731	Lung Blood	37	22-68
0.667	0.001	Muscle Ovary	12	34-70
0.611	0.291	Muscle Prostate	16	31-70
0.444	0.033	Muscle Testis	11	31-70
0.828	0.394	Muscle Blood	11	31-70
0.116	-0.239	Ovary Blood	9	42-66
0.476	0.083	Prostate Testis	39	22-70
0.844	0.121	Prostate Blood	33	22-70
0.681	0.244	Testis Blood	27	22-70

Supplementary Table 8. Correlation between tissue types for the EpiTOC clock.

Non-adjusted correlation	Age-adjusted correlation	Tissue type pair	Sample size	Chronological age range
0.072	-0.034	Breast Colon	15	21-70
0.191	0.046	Breast Kidney	5	42-66
-0.074	0.073	Breast Lung	16	37-70
-0.867	0.463	Breast Muscle	4	40-66
-0.086	0.318	Breast Ovary	28	21-69
0.844	0.833	Breast Blood	5	42-66
0.245	-0.035	Colon Kidney	38	36-70
0.161	0.031	Colon Lung	153	22-70
0.034	0.057	Colon Muscle	28	31-70
-0.162	-0.167	Colon Ovary	53	21-70
0.169	-0.039	Colon Prostate	85	22-70
0.287	-0.356	Colon Testis	38	22-70
0.413	0.191	Colon Blood	45	22-70
0.379	0.368	Kidney Lung	26	39-68
-0.008	-0.057	Kidney Muscle	12	42-70
0.471	-0.069	Kidney Ovary	11	42-68
0.178	0.109	Kidney Prostate	30	36-70
0.437	0.237	Kidney Testis	27	36-70
0.461	0.421	Kidney Blood	24	36-70
-0.009	0.044	Lung Muscle	25	31-67
0.176	0.401	Lung Ovary	51	22-70
0.134	0.101	Lung Prostate	73	22-70
-0.155	0.155	Lung Testis	33	25-68
0.541	0.531	Lung Blood	37	22-68
-0.168	0.953	Muscle Ovary	12	34-70
-0.249	0.071	Muscle Prostate	16	31-70
0.571	0.775	Muscle Testis	11	31-70
0.395	-0.087	Muscle Blood	11	31-70
0.245	0.073	Ovary Blood	9	42-66
-0.021	-0.082	Prostate Testis	39	22-70
0.218	0.092	Prostate Blood	33	22-70
-0.044	-0.038	Testis Blood	27	22-70

Supplementary Table 9. Correlation between tissue types for the Zhang clock.

Non-adjusted correlation	Age-adjusted correlation	Tissue type pair	Sample size	Chronological age range
0.662	0.043	Breast Colon	15	21-70
0.851	0.260	Breast Kidney	5	42-66
0.830	0.048	Breast Lung	16	37-70
0.988	0.882	Breast Muscle	4	40-66
0.777	0.447	Breast Ovary	28	21-69
0.723	-0.416	Breast Blood	5	42-66
0.785	0.246	Colon Kidney	38	36-70
0.713	0.185	Colon Lung	152	22-70
0.640	0.399	Colon Muscle	28	31-70
0.375	0.045	Colon Ovary	50	21-70
0.565	0.041	Colon Prostate	85	22-70
0.552	0.046	Colon Testis	38	22-70
0.855	0.097	Colon Blood	45	22-70
0.810	0.362	Kidney Lung	26	39-68
0.738	0.188	Kidney Muscle	12	42-70
0.451	-0.179	Kidney Ovary	11	42-68
0.590	0.172	Kidney Prostate	30	36-70
0.462	0.079	Kidney Testis	27	36-70
0.879	0.244	Kidney Blood	24	36-70
0.654	0.010	Lung Muscle	25	31-67
0.643	-0.268	Lung Ovary	48	22-70
0.491	-0.400	Lung Prostate	73	22-70
0.446	-0.066	Lung Testis	33	25-68
0.929	0.518	Lung Blood	37	22-68
0.550	0.753	Muscle Ovary	12	34-70
0.660	0.234	Muscle Prostate	16	31-70
0.467	0.110	Muscle Testis	11	31-70
0.830	-0.531	Muscle Blood	11	31-70
0.192	-0.790	Ovary Blood	9	42-66
0.523	0.167	Prostate Testis	38	22-70
0.556	-0.340	Prostate Blood	33	22-70
0.684	-0.253	Testis Blood	27	22-70

Supplementary Table 10. Correlation between tissue types for DunedinPACE clock estimates.

Non-adjusted correlation	Pair	Sample size	Chronological age range
0.011	Breast Colon	15	21-70
0.509	Breast Kidney	5	42-66
0.424	Breast Lung	16	37-70
0.526	Breast Muscle	4	40-66
0.298	Breast Ovary	28	21-69
0.336	Breast Blood	5	42-66
0.358	Colon Kidney	38	36-70
0.138	Colon Lung	153	22-70
0.332	Colon Muscle	28	31-70
-0.160	Colon Ovary	53	21-70
0.166	Colon Prostate	85	22-70
-0.174	Colon Testis	38	22-70
0.060	Colon Blood	45	22-70
0.433	Kidney Lung	26	39-68
0.181	Kidney Muscle	12	42-70
0.524	Kidney Ovary	11	42-68
0.351	Kidney Prostate	30	36-70
-0.471	Kidney Testis	27	36-70
0.448	Kidney Blood	24	36-70
0.026	Lung Muscle	25	31-67
-0.111	Lung Ovary	51	22-70
0.167	Lung Prostate	73	22-70
-0.300	Lung Testis	33	25-68
0.378	Lung Blood	37	22-68
0.540	Muscle Ovary	12	34-70
0.340	Muscle Prostate	16	31-70
-0.398	Muscle Testis	11	31-70
0.487	Muscle Blood	11	31-70
0.368	Ovary Blood	9	42-66
-0.234	Prostate Testis	39	22-70
0.308	Prostate Blood	33	22-70
-0.301	Testis Blood	27	22-70
