# Supplementary material for: Characterization of DNA methylation clock algorithms applied to diverse tissue types
Source: Aging (Albany NY). 2025 Jan 3;17(1):67–96. doi: 10.18632/aging.206182 (PMC11810061; doi:10.18632/aging.206182)
Supplement: Supplementary Tables 12-13 [file aging-17-206182-s004.pdf]

Supplementary Table 12. Linear regression of age acceleration on smoking, sex, BMI, and telomere length (non-sex specific tissues only).

	Blood		***Colon***		***Kidney***		***Lung***		***Muscle***	
**Observations**	***51***		***205***		***47***		***194***		***43***	
	Beta	*p*	*Beta*	*p*	*Beta*	*p*	*Beta*	*p*	*Beta*	*p*
**Horvath**										
**Smoking**	3.76	0.055	0.63	0.328	-4.13	**0.003**	-0.39	0.593	-3.34	**0.039**
**Sex**	2.43	0.250	0.14	0.820	-1.14	0.368	-0.13	0.857	-1.84	0.219
**BMI**	0.53	**0.009**	0.13	0.085	-0.32	0.034	0.17	**0.050**	0.09	0.581
**TQI**	1.26	0.738	-0.59	0.421	-4.33	**0.009**	-0.18	0.901	-0.45	0.825
**Hannum**										
**Smoking**	1.03	0.565	-3.43	0.106	-0.40	0.738	1.96	**0.005**	-1.83	0.152
**Sex**	1.36	0.488	-1.49	0.470	-0.33	0.775	0.01	0.991	-2.25	0.065
**BMI**	0.15	0.398	0.14	0.566	-0.07	0.609	0.12	0.138	0.07	0.596
**TQI**	-3.26	0.357	2.32	0.338	3.02	**0.044**	-0.54	0.688	-1.94	0.243
**EpiTOC**										
**Smoking**	0.00	0.838	-0.01	**0.027**	-0.00	0.599	0.01	**0.003**	-0.00	0.883
**Sex**	0.01	0.288	-0.02	**0.015**	0.00	0.397	0.00	0.787	-0.00	0.647
**BMI**	0.00	0.462	0.00	0.372	0.00	0.485	0.00	0.198	-0.00	0.949
**TQI**	-0.01	0.714	-0.01	0.303	0.00	0.851	-0.01	**0.020**	-0.00	0.388
**PhenoAge**										
**Smoking**	1.62	0.557	-3.53	0.200	-1.92	0.369	4.92	**<0.001**	-0.97	0.596
**Sex**	-0.26	0.931	1.05	0.694	-2.42	0.241	2.37	**0.024**	0.05	0.978
**BMI**	0.02	0.954	0.11	0.734	-0.05	0.834	-0.10	0.418	0.15	0.452
**TQI**	-1.26	0.815	5.64	0.074	6.02	**0.023**	-5.49	**0.009**	-1.45	0.544
**EpiClock**										
**Smoking**	3.82	0.026	-1.52	0.128	-0.98	0.454	2.00	**0.001**	-1.96	0.104
**Sex**	2.99	0.106	-1.20	0.215	-0.85	0.498	0.65	0.232	0.44	0.692
**BMI**	0.30	0.080	0.08	0.501	-0.01	0.967	0.07	0.284	0.14	0.285
**TQI**	0.54	0.868	0.19	0.869	-0.09	0.957	-1.85	0.095	-0.19	0.900
**AltumAge**										
**Smoking**	6.96	**0.024**	-0.69	0.557	-3.49	0.071	3.64	**0.002**	-2.22	0.313
**Sex**	4.15	0.209	1.88	0.099	0.77	0.674	1.40	0.218	0.23	0.911
**BMI**	0.70	**0.024**	0.29	**0.039**	-0.14	0.515	0.16	0.240	0.26	0.280
**TQI**	-1.05	0.858	-1.52	0.257	-3.76	0.107	-1.96	0.392	2.30	0.422
**Zhang**										
**Smoking**	-2.32	0.214	-2.18	**0.040**	-0.095	0.940	0.180	**0.004**	-3.82	0.086
**Sex**	3.12	0.126	0.166	0.871	-0.725	0.555	0.275	0.693	0.150	0.941
**BMI**	0.007	0.967	0.129	0.303	0.014	0.920	0.098	0.239	0.122	0.596
**TQI**	-14.3	**<0.001**	1.83	0.130	-1.62	0.296	-2.13	0.129	-0.202	0.942
**Pace**										
**Smoking**	0.009	0.898	0.064	**0.014**	0.420	0.224	0.083	**<0.001**	0.006	0.805
**Sex**	-0.034	0.664	0.002	0.938	-0.105	0.917	-0.029	0.088	0.045	**0.039**
**BMI**	-0.003	0.651	-0.001	0.632	0.001	0.722	-0.002	0.226	0.001	0.596
**TQI**	-0.173	**0.222**	-0.064	**0.030**	-0.091	**0.032**	0.486	0.150	0.038	0.201

The effect of sex is in reference to male and the effect of smoking is reference to non-smokers.

Supplementary Table 13. Linear regression of age acceleration on smoking, BMI, telomere length (sex specific tissues only).

	**Breast**		**Ovary**		**Prostate**		**Testis**	
**Observations**	**36**		**142**		**111**		**45**	
	Beta	*p*	*Beta*	*p*	*Beta*	*p*	*Beta*	*p*
**Horvath**								
**Smoking**	-0.39	0.841	1.29	0.078	-0.81	0.438	4.62	**0.032**
**BMI**	0.46	**0.049**	-0.06	0.511	0.16	0.225	-0.29	0.217
**TQI**	0.32	0.913	-0.68	0.591	-3.88	**0.023**	-3.55	**0.039**
**Hannum**								
**Smoking**	-2.28	0.073	-0.65	0.339	-1.39	0.234	1.23	0.279
**BMI**	0.41	**0.008**	0.02	0.775	0.26	0.075	-0.26	**0.045**
**TQI**	-0.31	0.868	0.97	0.405	-4.58	**0.017**	-1.88	**0.042**
**EpiTOC**								
**Smoking**	-0.01	0.241	-0.00	0.301	0.00	0.979	0.00	0.204
**BMI**	-0.00	0.856	-0.00	0.262	-0.00	0.862	0.00	0.546
**TQI**	0.01	0.431	0.00	0.669	-0.01	0.116	0.00	0.457
**PhenoAge**								
**Smoking**	-4.20	0.193	0.69	0.449	-0.38	0.838	1.94	0.205
**BMI**	0.56	0.139	-0.00	0.996	0.40	0.091	0.12	0.477
**TQI**	8.09	0.098	0.25	0.872	-4.84	0.110	-0.43	0.727
**EpiClock**								
**Smoking**	0.02	0.991	0.02	0.973	0.12	0.933	5.12	**0.037**
**BMI**	0.20	0.394	0.01	0.870	0.08	0.644	-0.27	0.327
**TQI**	3.82	0.213	1.48	0.145	-5.38	**0.017**	-5.54	**0.006**
**AltumAge**								
**Smoking**	-5.55	0.125	2.02	0.185	-1.27	0.354	6.82	0.103
**BMI**	0.44	0.296	-0.02	0.909	0.08	0.660	-0.50	0.286
**TQI**	0.76	0.886	1.04	0.692	-6.53	**0.004**	-6.38	0.059
**Zhang**								
**Smoking**	-1.32	0.339	0.009	0.995	-0.378	0.677	6.45	**0.034**
**BMI**	0.297	0.072	-0.194	0.154	0.196	0.088	-0.085	0.799
**TQI**	1.31	0.542	0.906	0.651	-4.38	0.004	-4.34	0.073
**Pace**								
**Smoking**	0.118	**0.003**	0.007	0.547	0.334	0.222	-0.008	0.588
**BMI**	0.008	0.082	0.001	0.518	-0.006	0.108	0.004	**0.032**
**TQI**	0.082	0.146	-0.009	0.669	-0.075	0.093	0.005	0.700

The effect of smoking is in reference to non-smoking.
